# From genes to modules, from cells to ecosystems

**DOI:** 10.15252/msb.202110726

**Published:** 2022-05-04

**Authors:** Tal Keidar Haran, Leeat Keren

**Affiliations:** ^1^ Department of Pathology Hadassah Medical Center Jerusalem Israel; ^2^ Department of Molecular Cell Biology Weizmann Institute of Science Rehovot Israel

**Keywords:** Cancer, Methods & Resources

## Abstract

Twenty years ago, molecular biology transitioned from predominantly studying genes as isolated elements to viewing them as part of complex modules, giving rise to the field of systems biology. This transition was made possible by technological advances that allowed to simultaneously measure the expression levels of thousands of genes in a single experiment and drove a shift toward analyses identifying gene sets, modules, and pathways involved in a biological process of interest. Today we are excitingly facing a similar turning point in cell biology, where single‐cell technologies have enabled us to approach cells as cellular modules.

## Graphical abstract

Similarly to genes, cells do not act in isolation but work in coordinated modules. This Commentary discusses a turning point in cell biology, where single‐cell technologies have enabled approaching cells as cellular modules.

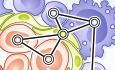

Cells are the basic units of life. The various functions within a cell and the coordination of processes between cells are performed by proteins, encoded by genes. Much of our understanding of biological processes has largely remained gene‐centric as historically research has focused on in‐depth characterization of single genes. The knowledge generated by gene‐focused research provides an invaluable foundation for both basic and translational research. Among the numerous examples of instrumental gene‐centric findings are the identification of the gene causing cystic fibrosis (Riordan *et al*, 1989) and the discovery of the receptor that facilitates the entry of SARS‐CoV‐2 into cells (Letko *et al*, 2020).

However, no gene or protein works in isolation. Just as a single mutation is not sufficient to explain most diseases, a single‐gene‐centered perspective fails to capture the breadth and complexity of functional processes and molecular mechanisms. For example, many protein subunits come together to form protein complexes, several different proteins facilitate metabolic reactions or regulatory processes, and jointly determine cellular phenotypes. Proteins and protein complexes are often organized in pathways introducing another level or regulatory complexity. In the late 1990s, microarrays allowed for the first time to measure the expression of many genes simultaneously, revealing the organization of genes into coordinated gene modules. In one of the first studies utilizing this technology, DNA microarrays were used to characterize the synchronized genome‐wide changes in gene expression occurring in response to a metabolic shift from fermentation to respiration in *Saccharomyces cerevisiae*. In this context, a gene module was defined as a set of genes showing a concordant change in their expression profiles under a given set of circumstances (DeRisi *et al*, 1997). Gene modules can also be defined based on existing knowledge, such as known metabolic or signaling pathways or protein–protein interactions. Once defined, these modules can be used as the basis for analyzing any biological question (Fig 1). Indeed, gene ontology enrichment, gene set enrichment analyses, and pathway analyses are routinely applied to various data types, (including genomics, transcriptomics, methylomics, chromatin accessibility, metabolomics, etc.), experimental setups, and biological questions. Analyzing gene modules reduces the complexity of the system and increases the pace of novel discoveries. It can lead to the identification of new functionalities for proteins that were previously not annotated via guilt by association and can facilitate the identification of upstream regulators, signaling hubs, and plausible resistance pathways to targeted therapies, such as in cancer.

**Figure 1:**
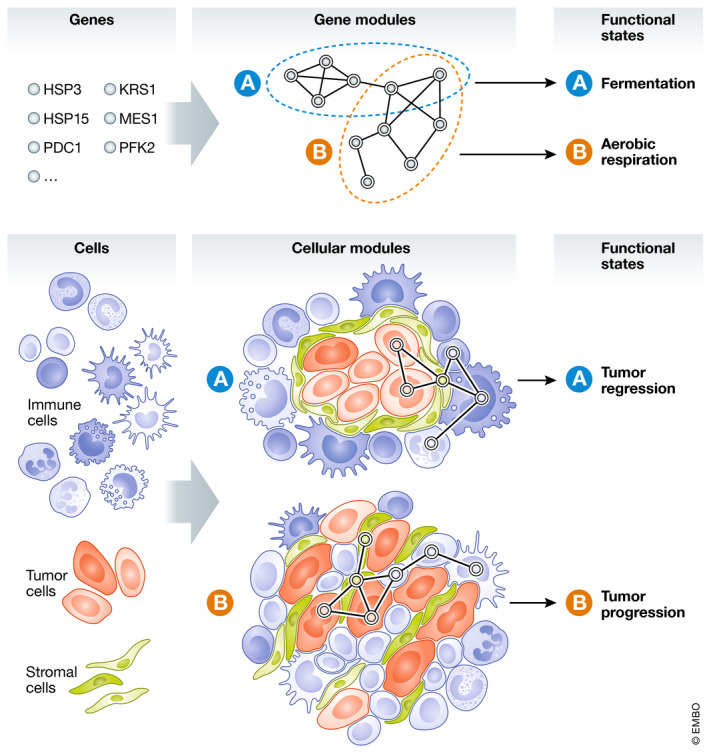
Genes modules and cellular ecosystems Top: a set of genes forms a gene module; members of a gene module are co‐expressed and jointly contribute to the resulting cellular phenotype or functional state. Bottom: a set of cells forms a cellular module, which can be defined both by its members and, as in the figure, by a unique spatial organization. Like genetic modules, distinct cellular modules propagate different functional states.

The last decade has seen a big shift from bulk analysis of tissues to analyses at the single‐cell level, focusing on extracting and analyzing individual cells, addressing cellular heterogeneity, and aiming at a precise characterization of diverse cell types and cell states. The development, improvement, and widespread adoption of single‐cell technologies have been key for enabling this shift. Such methods include single‐cell transcriptomics and proteomics, for example, by RNA sequencing (scRNAseq), Cytometry by Time of Flight (CyTOF), and single‐cell proteomics (SCP) and allow measuring expression profiles of individual cells within a large cell population. More recently, methods have been developed that allow performing detailed molecular profiling of cells while preserving spatial information. These include single‐molecule fluorescent in situ hybridization (smFISH), microdissection, in situ sequencing, cyclic fluorescence, and mass‐based imaging. These methods are well suited for identifying rare cell types, detecting tumor cells in blood or in a heterogenous tissue, and characterizing previously undescribed cell states.

The isolation and characterization of single cells at a high functional resolution is highly valuable, but it is still insufficient for understanding complex biological behaviors. Multicellular organisms are not simply a collection of cells. These cells communicate and organize into multicellular structures; they form tissues and organs. In cell biology, we are now at a point that may be comparable to single‐gene‐centered analyses. We are able to recognize and precisely characterize a variety of cell types and states in diverse experimental conditions. However, to a large extent, most analyses still approach these cells as isolated entities. For example, in oncology, numerous attempts have been made to identify a cell type or cell state that is predictive of response to checkpoint inhibitor immunotherapy. Among the many candidates, one can find cytotoxic effector T cells, peripheral memory T helper cells, and B cells (Huang & Zappasodi, 2022). While it may be plausible that a response is determined by a single cell type in a particular state, an alternative and rather compelling hypothesis is that the interaction of different cells in diverse states is what drives complex phenotypes such as a response to treatment. Drawing from experience in molecular biology, where grouping genes into functional modules allowed extracting meaningful information about responses at the molecular level, the logical next step in cell biology is to integrate single‐cell data into physiologically relevant ecosystems, the equivalent of cellular modules (Fig 1).

Indeed, research is beginning to emerge showing that cellular ecosystems exist and that these may mediate specific functions. In our work examining triple‐negative breast cancer using multiplexed imaging, we identified an interdependency between immune cell types present in the tumor microenvironment, where, for example, T cells were always accompanied by macrophages, but not the other way around (Keren *et al*, 2018). Leader *et al* (2021) used scRNAseq to identify a module consisting of activated T cells, IgG‐secreting plasma cells, and macrophages, with high prognostic value for the response of non‐small cell lung cancer (NSCLC) to immunotherapy. Cabrita *et al* (2020) found that tertiary lymphoid structures, comprising B cells, T helper cells, and naïve cytotoxic cells, improve response to immunotherapy and survival in melanoma. Recent studies using bulk sequencing and computational deconvolution were able to identify similar immune ecotypes in large pan‐cancer cohorts (Luca *et al*, 2021; Combes *et al*, 2022), indicating that such functional immune modules may be shared across tumor types. Reassuringly, prominent cellular modules are beginning to emerge, regardless of the technology used to identify them. The near future will undoubtedly bring further developments allowing finer‐grained resolution of cellular modules, and shed light on their distribution across individuals, age and in different disease states.

While the notion of cellular ecosystems is appealing, there are several challenges related to their identification and follow‐up analysis. To start with, outlining cellular modules analogous to gene modules requires that the cell types or cell states constituting each of these modules (e.g., “activated T cell” or “stem‐like memory T cell”) are clearly defined. However, the precise definition of a cell type or cell state is not as clear as that of a gene. What constitutes a cell state? Coarse delineations, such as those defined by the presence of cell surface markers (e.g., CD8, CD4, etc., as in the case of T lymphocytes), prove inadequate in view of the resolution achieved by single‐cell technologies, which has revealed great diversity within these classical cell‐type divisions. Taken to the extreme, heterogeneity is infinite, and each cell is unlike any other. Therefore, consistent definitions for cell subtypes and states are needed. Such efforts are underway, including establishing reference atlases for single‐cell transcriptomic data, from whole organism single‐cell atlases (Regev *et al*, 2017) to specialized atlases for individual cell types, for example, T cell subtypes.

Once cell types and states are defined, they may then be combined into modules. Genetic pathways are often defined based on various sources of complimentary information, encompassing, for example, the enzymes or protein kinases involved in a specific metabolic or signaling pathway respectively, or including the targets of a specific transcription factor. However, when analyzing cells instead of genes, due to our poor understanding of trans‐cellular interactions both *in vitro* and *in vivo*, the definition of cellular modules relies almost entirely on the co‐occurrence of specific cell states in a given experimental or natural condition. This is analogous to the identification of a family of genes that are up‐ or down‐regulated in response to a given perturbation, regardless of prior knowledge about their functional interrelationship. A key next step will be to set up experimental systems that allow to functionally interrogate these cellular ecosystems. Analogous to experiments where a particular gene is knocked out or over‐expressed to assess its functional role in a process, we need experimental systems that will allow accurate control of the composition and state of individual cells in the ecosystem. Notably, it is somewhat unclear what would be the relevant perturbations to such a system. Do we entirely remove a cell type? Do we alter the state of the cell? Or do we introduce a different cell type to the ecosystem? How do we discern between functions performed by different cells in the community? While we do not yet have answers for these complex questions, approaching a cellular system as an ecosystem of interacting cells and cell modules, influencing each other’s function, certainly offers exciting opportunities for “next‐generation” cell biology.

Altogether, it is becoming clear that if we want to understand a multicellular system, whether that is a cell population, an organoid, a tumor, or a whole organism, we need to take into account that similarly to genes, cells do not act alone but work in coordinated modules which remain to be precisely characterized and described. These are thrilling times as we start unraveling these cellular modules and envision experiments that will allow us to perturb these systems and understand their function.

## Disclosure and competing interests statement

The authors declare that they have no conflict of interest.
